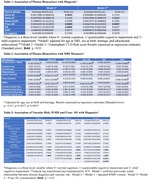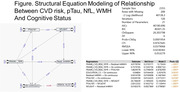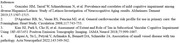# Vascular risk, WMH, pTau 181 and NfL, but not ApoE or Aβbeta 42/40, are associated with cognitive impairment in Latinos from the SOL‐INCA‐MRI study

**DOI:** 10.1002/alz.088342

**Published:** 2025-01-09

**Authors:** Charles Decarli, Shraddha Sapkota, Freddie Márquez, Wassim Tarraf, Carmen R Isasi, Richard B. Lipton, Linda C Gallo, Gregory A Talavera, Martha L Daviglus, Fernando Daniel Testai, Alberto R Ramos, Tatjana Rundek, Bharat Thyagarajan, Hector M Gonzalez

**Affiliations:** ^1^ University of California Davis, Davis, CA USA; ^2^ University of California, Davis, Davis, CA USA; ^3^ University of California, San Diego, La Jolla, CA USA; ^4^ Wayne State University, Detroit, MI USA; ^5^ Albert Einstein College of Medicine, Bronx, NY USA; ^6^ Department of Neurology, Albert Einstein College of Medicine, Bronx, NY USA; ^7^ San Diego State University, San Diego, CA USA; ^8^ University of Illinois at Chicago, Chicago, IL USA; ^9^ University of Illinois at Chicago, College of Medicine, Chicago, IL USA; ^10^ University of Miami Miller School of Medicine, Miami, FL USA; ^11^ Evelyn F. McKnight Brain Institute, Miami, FL USA; ^12^ University of Minnesota, Minneapolis, MN USA; ^13^ University of California San Diego, San Diego, CA USA

## Abstract

**Background:**

Serum AD biomarkers are becoming useful to the early and accurate diagnosis of neurodegenerative disease, but much of this work has been done with clinic‐based studies of mostly non‐Hispanic Whites. For this study, we examined relations between plasma biomarkers Aβ 42/40, pTau 181, NfL, GFAP, ApoE genotype and cognitive state in the SOL‐INCA‐MRI study. Given that prior work in SOL‐INCA found vascular risk to be associated with mild cognitive impairment^1^, we included vascular risk measured by the Framingham CVD risk score^2^ and white matter hyperintensity (WMH) burden from MRI as additional predictors.

**Method:**

2288 individuals enriched for cognitive impairment (29%), mean age 64.5 ± 6.8 years of which 69% were female and 60% had at least a high school education were studied. All subjects had MRI as well as plasma measures of Aβ 42/40, pTau 181, GFAP and NfL and a diagnosis of normal (71%), questionable (16%), or mild cognitive impairment (13%). Multi‐variate linear regression adjusted for age, sex, heritage, and education was used to build models of associations between each of the measures and cognition. Finaly, structural equation modeling was used to assess the structural relationship between measured variables.

**Result:**

Higher serum pTau181 and Nfl concentrations were significantly associated with diagnosis (Table 1). PTau181, NfL and GFAP were also associated with MRI measures, particularly WMH (Table 2). CVD risk also was significantly associated with pTau181 (β=1.23 ± 0.34, p <0.0001) and NfL (β=25.4 ± 2.8, p <0.0001). In a final model (Table 3), sex, CVD risk, WMH and pTau181 remained significantly associated with cognition. Best fit SEM summarizes the relationship of these measures to each other and cognition (Figure). ApoE4 genotype and Abeta 42/40 ratio were not associated with diagnosis in this group.

**Conclusion:**

In a group of Latinos, enriched for cognitive impairment, vascular risk, WMH and pTau181 were significantly associated with diagnosis. The lack of association with serum Abeta 42/40 ratio suggest that vascular disease and not AD pathology are most strongly associated with cognition. The relationship between CVD risk, WMH, and pTau181is consistent with emerging evidence relating tau phosphorylation to vascular disease^3, 4^.